# The impact of chronic intermittent hypoxia on hematopoiesis and the bone marrow microenvironment

**DOI:** 10.1007/s00424-016-1797-6

**Published:** 2016-02-09

**Authors:** Inês Alvarez-Martins, Leonor Remédio, Inês Matias, Lucília N. Diogo, Emília C. Monteiro, Sérgio Dias

**Affiliations:** Angiogenesis Lab, CIPM, Portuguese Institute of Oncology, IPOLFG, EPE, Lisbon, Portugal; Instituto de Medicina Molecular, Edificio Egas Moniz, Faculdade de Medicina da Universidade de Lisboa, 1649-028 Lisbon, Portugal; CEDOC, Nova Medical School, Universidade Nova de Lisboa, Campo dos Mártires da Pátria, 130, 1169-056 Lisbon, Portugal; Translational Pharmacology Lab, Universidade Nova de Lisboa, Campo dos Mártires da Pátria, 130, 1169-056 Lisbon, Portugal

**Keywords:** Chronic intermittent hypoxia, Hematopoiesis, Vascular niche, Bone marrow microenvironment

## Abstract

Obstructive sleep apnea (OSA) is a highly prevalent sleep-related breathing disorder which is associated with patient morbidity and an elevated risk of developing hypertension and cardiovascular diseases. There is ample evidence for the involvement of bone marrow (BM) cells in the pathophysiology of cardiovascular diseases but a connection between OSA and modulation of the BM microenvironment had not been established. Here, we studied how chronic intermittent hypoxia (CIH) affected hematopoiesis and the BM microenvironment, in a rat model of OSA. We show that CIH followed by normoxia increases the bone marrow hypoxic area, increases the number of multipotent hematopoietic progenitors (CFU assay), promotes erythropoiesis, and increases monocyte counts. In the BM microenvironment of CIH-subjected animals, the number of VE-cadherin-expressing blood vessels, particularly sinusoids, increased, accompanied by increased smooth muscle cell coverage, while vWF-positive vessels decreased. Molecularly, we investigated the expression of endothelial cell-derived genes (angiocrine factors) that could explain the cellular phenotypes. Accordingly, we observed an increase in colony-stimulating factor 1, vascular endothelium growth factor, delta-like 4, and angiopoietin-1 expression. Our data shows that CIH induces vascular remodeling in the BM microenvironment, which modulates hematopoiesis, increasing erythropoiesis, and circulating monocytes. Our study reveals for the first time the effect of CIH in hematopoiesis and suggests that hematopoietic changes may occur in OSA patients.

**Electronic supplementary material:**

The online version of this article (doi:10.1007/s00424-016-1797-6) contains supplementary material, which is available to authorized users.

## Introduction

Hematopoiesis has long been known to be affected by environmental hypoxia [64]. Despite numerous reports relating hypoxia with hematopoietic modulation [3, 5, 44, 46, 48, 49, 61, 64] and the great attention currently given to hypoxia-inducible factor (HIF) [34], the current scientific knowledge largely relies on of studies performed under acute hypoxia stimulation in isolated systems. Therefore, the role of chronic systemic hypoxia in the bone marrow (BM) microenvironment and hematopoiesis is still unknown.

Here, we studied the role of environmental hypoxia using a clinically relevant chronic intermittent hypoxia (CIH) model, which consists of exposing the experimental animals to a paradigm of CIH for 30–35 days, as a model of obstructive sleep apnea syndrome (OSA) [25]. OSA is an increasingly prevalent condition affecting children and adults, which is renowned as a frequent secondary cause of hypertension [16]. There is ample evidence for the involvement of BM-derived cells in the pathophysiology of hypertension [78] but a relation between OSA and modulation of the BM microenvironment had not been shown and is the subject of the present study. The clinical hematological aspects of OSA are still largely unknown, with several studies reporting mainly platelet activation and increased hematocrit, but not assessing BM or circulating blood cell alterations [22]. Furthermore, these studies typically compared groups of patients with differing disease phenotypes [32] or treated versus untreated patients [42] but not healthy controls versus patients and do not explore the mechanisms involved [22].

Mechanistically, we focused on the vascular compartment of the BM, because it mediates the differentiation and proliferation of hematopoietic cells, as well as their egress from the BM microenvironment [40]. Furthermore, one of the most striking effects of hypoxia is the promotion of angiogenesis, which results from hypoxia inducible factor (HIF)-mediated increase in vascular endothelial growth factor (VEGF) expression [62].

The causal relationship between intermittent hypoxia and increased VEGF expression is known in both OSA patients [43] and healthy volunteers [7], but the relevance of this finding is still unknown.

The present work aims to explore for the first time whether CIH induce changes in the BM vascular compartment, which might in turn modulate hematopoiesis.

Our data suggests that CIH may promote erythropoiesis, increase the multipotential progenitor cell-derived CFUs accompanied by an increase in BM myeloid and B lymphocyte counts and a decrease BM T cells. Additionally, CIH also induces expansion of the blood monocyte compartment and perturbs the BM microenvironment by interfering with the vascular niche. Together, our results reveal hematopoietic and hematological complications of CIH which need to be validated and evaluated in a clinical setting.

## Methods

### Animals

Experiments were performed in twelve (12) male Wistar rats, aged 8–12 weeks, obtained from the NOVA Medical School animal facility. Animals were housed in polycarbonate cages, under 12-h light/dark cycles (8 am–8 pm) at a room temperature 22 ± 2.0 °C and relative humidity 60 ± 10 %. Rats were maintained one or two per cage with ad libitum access to food and water. Applicable institutional and governmental regulations concerning ethical use of animals were followed, according to the NIH Principles of Laboratory Animal Care (NIH Publication 85–23, revised 1985), the European guidelines for the protection of animals used for scientific purposes (European Union Directive 2010/63/EU), and the Portuguese Law n° 113/2013. Experimental procedures were previously approved (nr. 21/2013/CEFCM) by the Institutional Ethics Committee of the NOVA Medical School for animal care and use in research.

### In vivo experiments

Rats were divided into two groups: normoxia and CIH. Animals were kept in a eucapnic atmosphere, inside of medium A-chambers (76 × 51 × 51 cm, A-60274-P, Biospherix Ltd, NY, USA) with ad libitum food and water access. The chambers were equipped with gas injectors and sensors for oxygen (O_2_) and carbon dioxide (CO_2_) levels in order to ensure the accuracy of CIH cycles. Accumulation of CO_2_ was prevented by the continuous flow of the gas mixtures through vent holes and the presence in the chamber of self-indicating soda lime, which absorbs the expired CO_2_. The CO_2_ levels inside the chambers never exceeded 1 %. A silica gel container was also placed inside the chambers in order to absorb water. Oxygen concentration inside the chambers was controlled using 100 % nitrogen (N_2_) and 100 % O_2_ by an electronically regulated solenoid switches in a three-channel gas mixer, which gradually lowered oxygen in the chamber from 21 to 5 % O_2_ (OxyCycler AT series, Biospherix Ltd, NY, USA). The chambers were infused with 100 % N_2_ for 3.5 min to briefly reduce the O_2_ concentration to 5 % and then with 100 % O_2_ for 7 min to restore oxygen to ambient levels of 21 %, until the start of the next CIH cycle. Each CIH cycle lasted 10.5 min, and rats were exposed during their sleep period (light phase of light/dark cycle) to 5.6 CIH cycles/h, 10.5 h/day for 32 days and analyzed 3 days after the hypoxic period. During the remaining hours of the day, the chambers were ventilated with a constant flow of room air to keep oxygen levels at 21 %. O_2_ was purchased as regular gas bottles (Gasin, Portugal), while N_2_ was generated from the air by pressure swing adsorption technology using a high-output nitrogen generator (Nitrogen 15 Plus, PSA Technology, Sysadvance, Maia, Portugal).

### Sample collection

After exposing rats to 32 days of hypoxia followed by 3 days in normoxia, rats were sacrificed by intraperitoneal injection with medetomidine (0.5 mg/kg body weight, Domitor®, Pfizer Animal Health) and ketamine (75 mg/kg body weight, Imalgene 1000®, Mérial, Lyon, France), and cardiac puncture was performed to collect peripheral blood. Blood was collected in EDTA-coated tubes (Multivette 600, Sarstedt), and plasma sampling and complete blood counts were performed. Femur BM cells were flushed out with PBS 2 % FBS, and the total BM cell count was assessed using a Burker hemocytometer (Blau Brand).

### Hypoxia quantification in bone marrow sections

Three days after the hypoxic period, the subjects were intravenously injected (via tail vein) with 60-mg/kg pimonidazole hydrochloride (Hypoxyprobe, Inc, Burlington, USA), a misonidazole-based compound, which forms adducts with thiol groups of proteins, peptides, and amino acids specifically in hypoxic cells (pO_2_ < 10 mmHg) (81). Two hours later, rats were euthanized using 60-mg/kg sodium pentobarbital IV (Eutasil, Ceva Santé Animale, Libourne, France) and transcardially perfused with PBS. Quantification of BM hypoxic areas was performed using the ImageJ software in 10 high-power fields (×400 magnification) per animal.

### Flow cytometry

Bone marrow and peripheral blood cells were treated with red blood cell lysis buffer (Biolegend) for 15 min in the dark and were then stained for anti-CD90 (HIS51) fluorescein isothiocyanate (FITC), anti-CD11b (WT.5) allophycocyanin (APC) (BD Biosciences), anti-CD117 (2B8) APC, anti-CD19 (1D3) PE-cyanine 7 (PE/Cy7) (eBiosciences), and anti-CD3 (17A2) APC-cyanine 7 (APC/Cy7) (BioLegend). Flow cytometric analyses were carried out using an LSR Fortessa flow cytometer equipped with FACS Diva 6.2 Software (BD Biosciences). Data were analyzed with a FlowJo 9.8.2 software.

### In vitro colony forming assay

Femur BM cells were flushed with PBS 2 mM EDTA, treated with red blood cell lysis buffer (Biolegend) for 15 min in the dark and plated onto petri dishes for 2 h. Non-adherent cells (10^5^ cells) were collected and plated onto a semi-solid cytokine-supplemented methylcellulose medium (MethoCult GF M3434) (Stemcell Technologies). Each colony formed in this semi-solid medium is single-cell derived and represents the identity of the original progenitor cell [6, 15]. The resulting colonies were scored after 1–2 weeks of culture, according to manufacturer’s instructions.

### Immunostaining and imaging

Femurs were formalin-fixed, decalcified with formic acid for 3 days, and processed for routine histopathology. Immunohistochemistry staining was performed on 3-μm slices. Sections were treated for antigen retrieval and incubated with the primary antibodies listed in Table 1 for 1 h at room temperature, immunostained according to the visualization system manufacturer’s instructions and counterstained with hematoxylin. The slides were then analyzed using a Leica DM2500 microscope, and all images were acquired with the 40× objective. The number of vessels or cells stained by each marker was quantified as a mean of 10 representative images of individual rat femurs. Sections for immunofluorescence were incubated with VE-cadherin for 1 h at room temperature followed by an incubation with an Alexa Fluor 488 secondary antibody (Life Technologies). DNA was stained with DAPI Vectashield mounting medium (H-1200, Vector Laboratories). Imaging was performed using a Zeiss LSM 510 META microscope, and images were acquired with the 40× water immersion objective.

**Table 1:** Primary antibodies used for immunohistochemistry

Antigen	Antigen retrieval	Dilution	Brand
CD105 (Endoglin)	HIER, Tris-EDTA pH 9	1:150	R&D AF1320
CD11b	HIER, Tris-EDTA pH 9	1:100	BD 550282
SMA	HIER, Tris-EDTA pH 9	1:500	DAKO HHF35
VE-cadherin	PIER, Pepsin	1:150	R&D AF1002
vWF	PIER, Pepsin	1:300	DAKO A0082
Anti-goat, peroxidase		ready-to-use	VectorLabs MP-7405
Anti-mouse, peroxidase		ready-to-use	DAKO K4007
Anti-rabbit, peroxidase		ready-to-use	DAKO K4011
Anti-rat, peroxidase		ready-to-use	VectorLabs MP-7444

### RNA isolation and quantitative PCR

Bone marrow cells were collected by flushing off tibias with PBS 2 % FBS. Cells were centrifuged at 1200 rpm for 5 min, collected to TRIzol Reagent (Invitrogen), and RNA was extracted according to manufacturer’s instructions. Reverse transcription was performed with SuperScript II (Invitrogen), according to the manufacturer’s protocol. Quantitative PCR was performed with Power SYBR Green PCR Master Mix (Roche) on a ViiA^TM^ 7 Real-Time PCR System (Life Technologies). The sequences of the oligonucleotides used are included in Table 2. A primer concentration of 180 nM was found to be optimal in all cases. Amplification of hypoxanthine guanine phosphoribosyl transferase (*Hprt*) was used for sample normalization.

**Table 2:** List of primers used for RT-PCR

Gene	Forward primer (5′-3′)	Reverse primer (5′-3′)
*rHPRT*	GACCGCTTTTCCCGCGAGCC	TCACGACGCTGGGACTGAGGG
*rAdm*	ACCGCACGGCTCGACACTTC	TCCCACGACTTAGCGCCCAC
*rAngpt1*	TGATGCCTGTGGCCCTTCCA	CATGGTTTTGCCCCGCAGTGT
*rAngpt2*	TGTCCGGCGAGGAGTCCAAC	GATTTTGCCCGCCGTGCCTG
*rBmp4*	AGTTTGTTCAAGATTGGCTCCC	CGACCATCAGCATTCGGTTA
*rCdh2*	TCTGCACCAGGTTTGGAATGGGT	ACATACGTCCCAGGCTTTGATCCC
*rCsf1*	GCCACCGAGAGGCTACAGGAA	TTTGGACACAGGCCTCGTTCTGTT
*rCsf2*	GGTCTACGGGGCAACCTCACC	AGTTTCCGGGGTTGGAGGGCA
*rCsf3*	CCTCGGGGTGGCCCCTACTG	CCCGACGCTGGAAGGCAGAA
*rCxcl12*	GCATCAGTGACGGTAAGCCA	TCTCAAAGAATCGGCAGGGG
*rCxcr4*	TGGAGAGCGAGCATTGCC	GCAGGGTTCCTTGTTGGAGT
*rDhh*	TCCCCAACTACAACCCCGA	GCTAGAGCATTCACCCGCTC
*rDkk1*	CTCTATGAGGGCGGGAACAA	GCAAGGGTAGGGCTGGTAGT
*rDll1*	TCTCCTGACGACCTCGCAACA	GGTGCCTCTGTGTGGTCAGGC
*rDll4*	CTGGCCGGGAACCTTCTCACTC	TCTCTGGCCGCAGGTCGTCTC
*rEpo*	CCCTATTTACGGGGTGCTGG	CTGTCTCTGCCCCTGAGTTC
*rFgf1*	AGGGACAGGAGCGACCAGCA	TACACTTCGCCCGCGCTTTCC
*rFgf2*	TCCGGGAGAAGAGCGACCCA	CCGGTTCGCACACACTCCCTTG
*rFlt3l*	AGCTCTGAAGCCCTGTATCGGGA	ACTGCACCTCCAGGCACCGA
*rFlt4*	CCCTGCTTGGTGTCCATTCC	GTCGTCCCACAACACCTCC
*rHes1*	TCAACACGACACCGGACAA	GCTTTGATGACTTTCTGTGCT
*rHey1*	GCCGACGAGACCGAATCAA	TTCGCAGATCCCTGCTTCTC
*rHey2*	CCCTTGCGAGGAGACGACCT	GCTCCCCACGTCGATGGTCT
*rHif1α*	GCTTACACACAGAAATGGCCC	GTCCTCCCCCGGCTTGTTAG
*rHif2α*	CCGCCTCATGTCTCCATGTT	CAGCTTGTTGGACAGGGCTA
*rIgf1*	CTTTGCGGGGCTGAGCTGGT	AGCCCCTTGGTCCACACACGAA
*rIgfbp3*	AAGGCGCTGCTGAATGGCCG	GCTGGGAGGGGAGGTAGGCA
*rIgfbp5*	ACCTGCCCAACTGTGACCGC	GGCCACGAGAAGGCTTGCACT
*rIl3*	TGATGCTCTTCCACCAGGGACT	AGTCCTGCAATCCAACGTCCTGA
*rIl6*	CTCTCCGCAAGAGACTTCCAGC	AGGGAAGGCAGTGGCTGTCAA
*rIl11*	CCGACTGGAACGGCTACTTC	CAAGGCTAGGCGAGACATCAAG
*rJag1*	GGAAGGCTGGATGGGTCCTGA	TGCAGGAGCCATGCTTGGGA
*rJag2*	CGGGCCTCGTCGTCATTCCCT	CAGGCCTCCACGATGAGGGTGA
*rKdr*	CGGTCATCCTCACCAATCCC	CCGATCTGGGGTGGAACATT
*rKitl*	ACAAAACTGGTGGCGAATCTTCCAA	TCCCGGCGACATAGTTGAGGGT
*rPecam1*	TGGCTTGAGTGGGCGGATGG	AGCCGGGTGGCTGAGGGAAG
*rSmad2*	TGTGCAGAGCCCCAACTGTAACCA	GGATTTTGCACACTGTCGCGGG
*rSmad3*	AGGCCATCACCACGCAGAACG	AGCCGGCCATCCAGTGACCT
*rTgfb1*	AGCCCGAGGCGGACTACTAC	TGCGTTGTTGCGGTCCACCATT
*rThpo*	TGTCCCCACCCCACTCTGTGC	GTGTGGGGCCTCTCCCCTGA
*rVcam1*	CGGAGCCTCAACGGTACTTTGG	GCGAGCGTTTTGTATTCAGGGGA
*rVegfa*	GCACTGGACCCTGGCTTTAC	TCTGCTCCCCTTCTGTCGT

### Statistical analysis

Results are expressed as mean ± standard deviation. Data were analyzed using unpaired two-tailed student’s *t* test. *P* values of <0.05 were considered statistically significant.

## Results

### Chronic intermittent hypoxia does not affect BM cell number but modulates/perturbs hematopoiesis

**Fig. 1:**
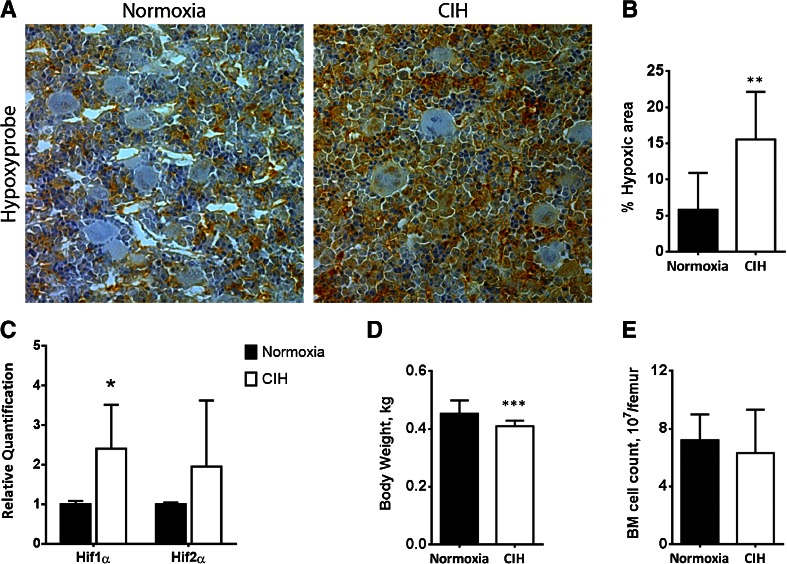
Chronic intermittent hypoxia affects the hypoxic state of the bone marrow and decreases body weight but does not affect bone marrow cell counts. **a** The extent of BM hypoxia was increased in animals exposed to CIH, as assessed by pimonidazole staining and **b** the significant increase in hypoxic area in CIH animals. **c** CIH also have increased *Hif1α* expression. **d** Rats subjected to CIH had a lower body weight than controls. **e** Total BM cell count shows that CIH does not modify BM cellularity. Results are represented as the mean ± SD of bone marrow sections from six male Wistar rats (*p < 0.05; **p < 0.01)

**Fig. 2:**
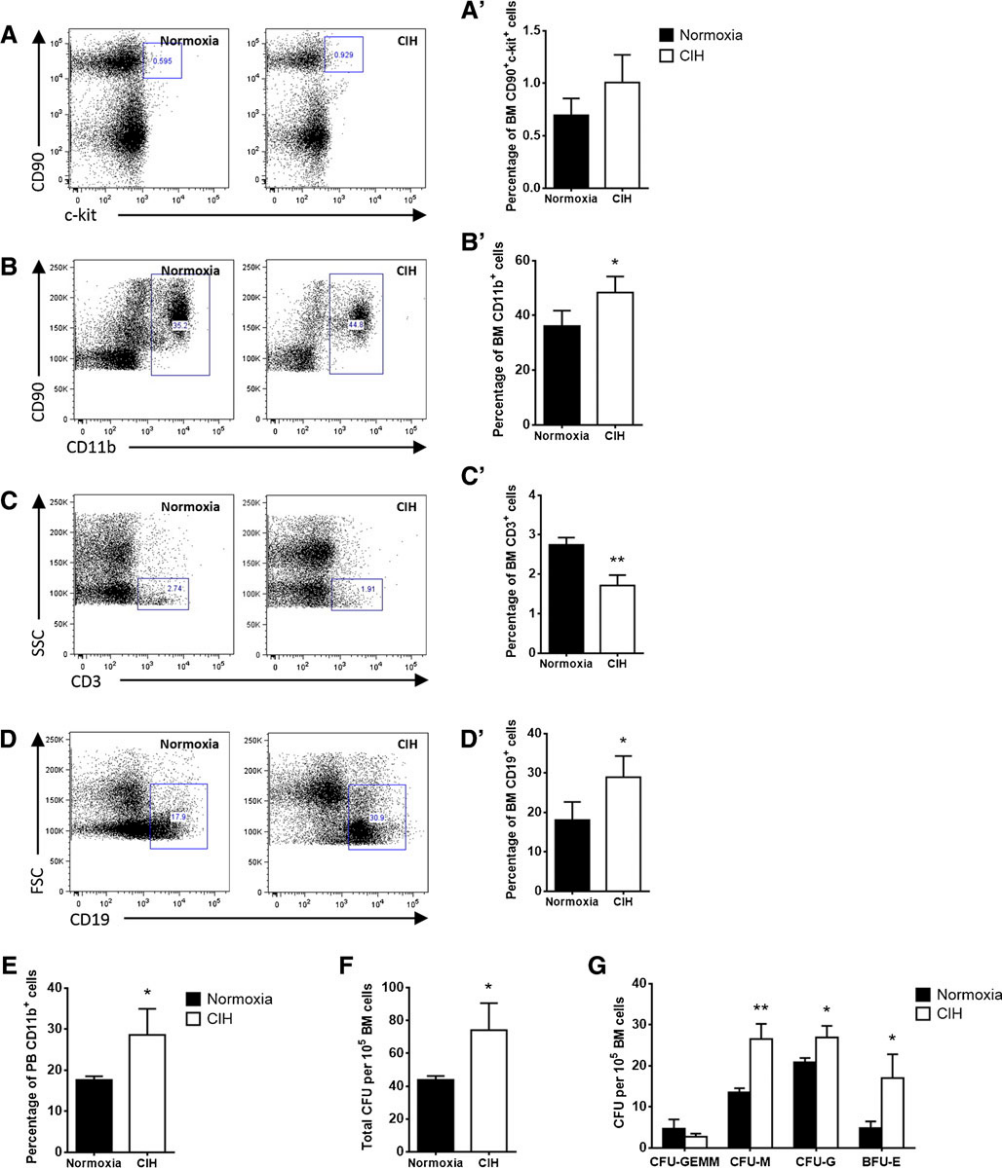
Rats exposure to chronic intermittent hypoxia affects specific hematopoietic lineages and the commitment of bone marrow progenitor cells. **a**–**d** Representative plots of the flow cytometric analysis of bone marrow cells from normoxic (*n* = 5) and CIH (*n* = 5) exposed rats. Quantification of **a’** CD90+/c-kit + stem and progenitor cells did not reveal a significant alteration in CIH animals. However, **b’** CD11b^+^ myeloid- and **d’** CD19^+^ B cells were increased as opposed to **(c’)** CD3 T lymphocytes that decreased upon CIH exposure. **e** Quantification of peripheral blood CD11b^+^ cells by flow cytometry also revealed an increase in the percentage of those cells in circulation. **f** Colony-forming unit counts from methylcellulose culture of 10^5^ BM cells reveal that CIH treatment induces an increased the number of HSPC, **g** with a particular increase in macrophage, granulocyte and erythroid (CFU-M, CFU-G and BFU-E) colonies. Results are represented as the mean ± SD of bone marrow cells from five male Wistar rats (*p < 0.05; **p < 0.01)

In this study, six male Wistar rats were exposed to chronic intermittent hypoxia for 32 days and then left in normoxia for three more days. The post-hypoxic period before the analysis allowed us to observe the persistent changes in hematopoiesis and the BM microenvironment. Notably, as assessed by hypoxyprobe staining, the extent of BM hypoxia was increased in CIH exposed animals (Fig. 1a, b), which was accompanied by an upregulation in *Hif1a* messenger RNA (mRNA) (Fig. 1c), CIH was also associated with a significant decrease in whole body weight (Fig. 1d), an observation that had already been associated with both sustained and intermittent hypoxia exposure [45, 76]. Nevertheless, concerning the BM cellular content (corrected to the total body weight of the animals), there was no alteration in the total number of BM cells caused by CIH (Fig. 1e). However, the percentage of specific hematopoietic lineages within the BM changed. Specifically, we found an increase in the CD11b^+^ myeloid cells (the majority of which are monocytes) in hypoxia-exposed animals (from 36.10 ± 5.60 to 48.38 ± 5.86 %), and a modulation in the lymphoid compartment, with a significant increase in CD19^+^ B cells (18.07 ± 4.55 % in normoxia and 28.90 ± 5.40 % in CIH) and a decrease in the percentage of CD3^+^ T lymphocytes (2.74 ± 0.18 and 1.70 ± 0.26 % in normoxia and CIH exposed rats, respectively) (Fig. 2b–d). Although we did not observe a significant variation in the percentage of CD90^+^/c-kit^+^ stem and progenitor cells by flow cytometry (Fig. 2a and a’), in vitro colony-forming units (CFU) assays revealed an increase in 1.5-fold in the total number of CFUs in animals from the CIH group (Fig. 2f). Such assays allowed us to identify and count single-cell derived colonies, representing either multipotent (CFU-granulocyte-erythrocyte-macrophage-megakaryocyte, CFU-GEMM) or monopotent (CFU-monocyte, CFU-M; CFU-granulocyte, CFU-G or bursting forming units-erythrocyte, BFU-E) progenitors. Our data show that chronic intermittent hypoxia treatment significantly increased CFU-M, CFU-G and BFU-E colony numbers, without significant alterations in the multipotent capacity (CFU-GEMM) of treated rats (Fig. 2g). Although these results suggest CIH favors the expansion of monopotent progenitors, we cannot rule out the possibility that the monopotent progenitors arose from more primitive multipotent progenitors.

**Fig. 3:**
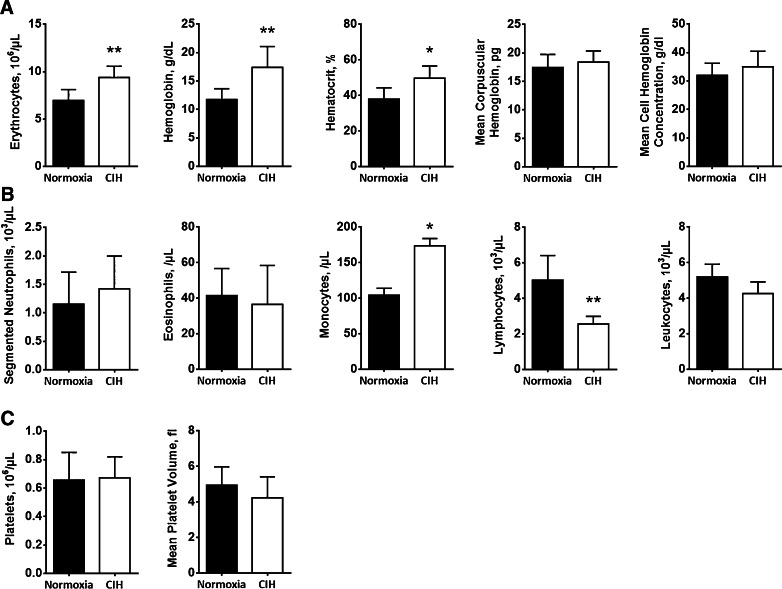
Chronic intermittent hypoxia modulates circulating blood counts. **a** CIH may promote erythropoiesis. Erythrocyte, hemoglobin, and hematocrit, as well as mean corpuscular hemoglobin and mean cell hemoglobin concentration were assessed by peripheral blood cell counts. The erythrocyte count, hemoglobin, and hematocrit in CIH-exposed rats (*n* = 5) are significantly different from those in normoxia (*n* = 5) (*p < 0.05). **b** CIH increases circulating monocytes and decrease lymphocytes. However, peripheral blood cell counts showed no differences in neutrophils, eosinophils, or leukocytes. **c** Platelet count and mean platelet volume are not modified by exposure to CIH. Results are represented as the mean ± SD of blood samples from five male Wistar rats (*p < 0.05; **p < 0.01)

Having characterized the changes in BM hematopoietic lineages accounted to CIH exposure, we asked whether these alterations could also be identified in peripheral blood (PB). Indeed, as shown in Fig. 3, we observed a significant increase in erythrocyte counts, in hemoglobin and in the hematocrit of CIH exposed rats (Fig. 3a). Our data also shows a decrease in circulating lymphocytes and an increase in monocytes upon CIH exposure (Fig. 3b), a finding that was also confirmed by flow cytometry, showing increased CD11b^+^ myeloid cells in the PB (Fig. 2e). In contrast, leukocyte and granulocyte (eosinophil and neutrophil) counts were not affected by CIH. Platelet counts and mean platelet volume were also similar in the CIH and normoxia groups (Fig. 3c).

Our data thus mimic some of the clinical aspects observed in OSA patients, as we observed no changes in platelet counts, but fails to reproduce other symptoms, such as the increase in platelet activation and aggregation, as assessed by mean platelet volume. Moreover, we report for the first time an increase in the myeloid compartment, both the BM and PB and a modulation in the percentage of B and T lymphocytes in the BM of animals exposed to CIH.

### Chronic intermittent hypoxia affects the bone marrow vasculature and modulates monocyte counts

**Fig. 4:**
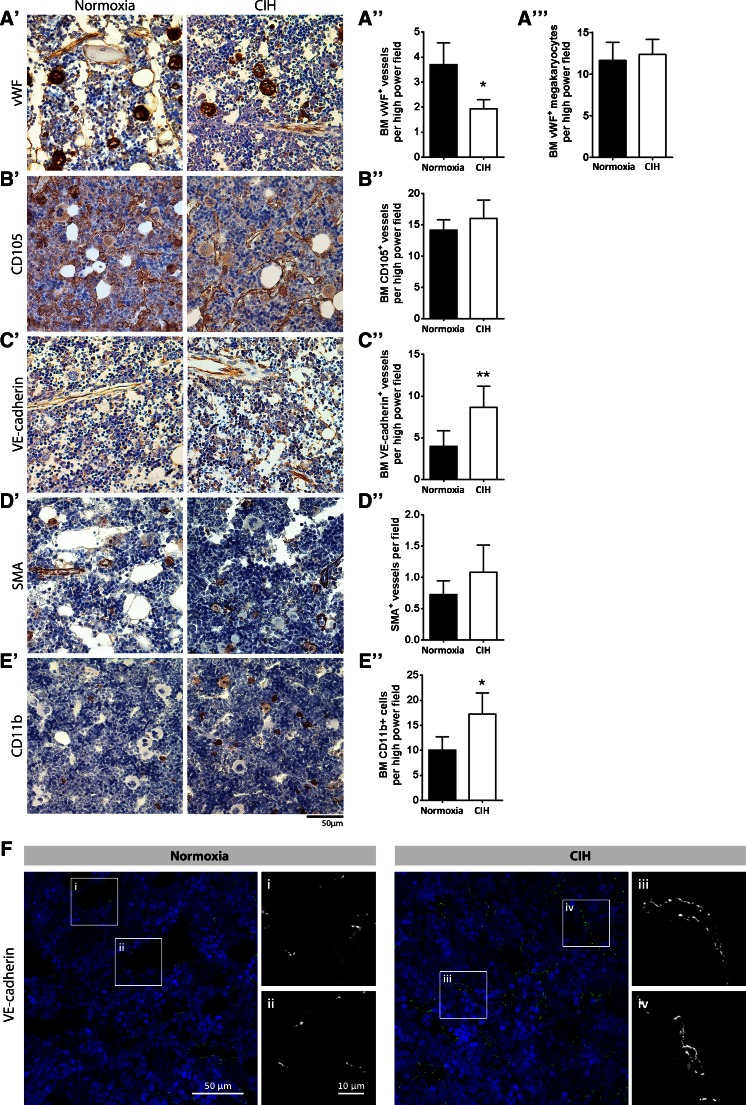
Chronic intermittent hypoxia modifies the BM vascular structure. **a’–e’** Representative images of femur bone marrow stained with vWF, CD105, VE-cadherin, SMA, and CD11b counterstained with hematoxylin. **a”, c”, d”** BM from CIH exposed rats (*n* = 6) has more VE-cadherin^+^ vessels and SMA coverage but less vWF^+^ sinusoids (400×, Leica DM2500). **e’, e”** Representative images of CD11b immunohistochemistry in femur BM show an increase in BM monocyte count in CIH exposed animals. (400×, Leica DM2500) **a’, a”’, b’, b”** No changes in the total number of vessels or in megakaryocyte count were observed, as accounted by CD105 and vWF staining, respectively. Results are represented as the mean ± SD of bone marrow sections from six male Wistar rats (*p < 0.05; **p < 0.01). **f** Representative images of femur bone marrow fluorescently immunostained for VE-cadherin show an increase in total VE-cadherin vessels and in VE-cadherin vessel coverage. Scale bar, 50 μm (insets magnified 2.5×). Images were acquired with a Zeiss LSM 510 META microscope

Having shown that exposure of rats to CIH perturbs hematopoiesis, as evidenced by changes in circulating mononuclear cell and erythrocyte counts, next we sought to characterize the phenotypic and molecular alterations that occurred in the BM microenvironment that could account for such changes. As shown in Fig. 4, the BM vasculature of CIH exposed animals suffered “phenotypic alterations,” as shown by the significant increase in VE-cadherin expressing vessels (Fig. 4c’, c”) and their VE-cadherin coverage (Fig. 4f), the increase in smooth muscle cell coverage (Fig. 4d’, d”), and the decrease in the number of vessels that were positive for vWF (Fig. 4a’, a”). In contrast, endoglin (CD105)-expressing vessels did not vary upon CIH exposure (Fig. 4b’, b”). Similarly, the BM megakaryocyte content (also assessed by vWF staining) did not vary with CIH exposure (Fig. 4a’, a”’).

To demonstrate that there was undoubtedly an increased expansion of myeloid cells within the BM, we assessed the BM CD11b^+^ cell (monocyte) content. In accordance with the flow cytometric and complete blood count data, we observed an increment in the number of BM monocytes in CIH treated animals (Fig. 4e’, e”).

Together, these data show the BM vasculature and in particular the VE-cadherin and vWF-expressing vessels, and the pericyte/smooth muscle cell vessel coverage are affected by CIH exposure. This morphological change in BM vessels of CIH-treated animals is accompanied by a significant increase in the number of CD11b + monocytes.

### Chronic intermittent hypoxia modulates the expression of “angiocrine genes”

**Fig. 5:**
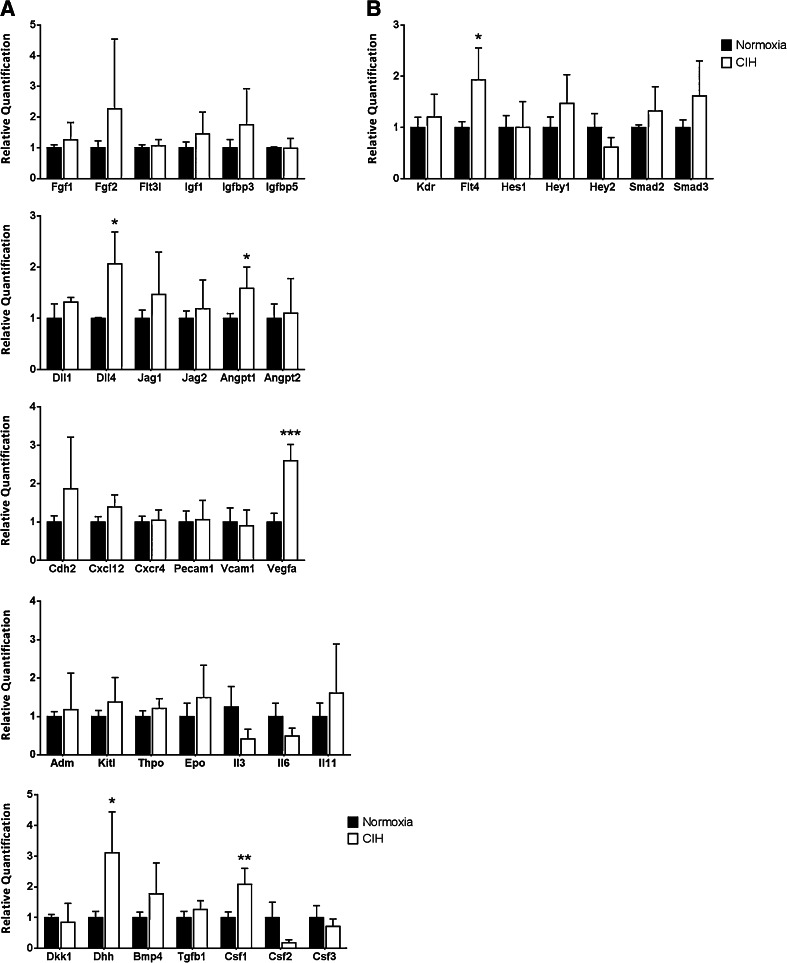
Chronic intermittent hypoxia modulates bone marrow angiocrine gene expression. **a** Angiocrine gene modulation was assessed by relative quantification of mRNA of total BM samples from normoxia (*n* = 6) and CIH (*n* = 6) treated rats. As determined by RT-PCR, we observed an increase in *Vegfa*, *Dll4*, *Angpt1*, *Dhh*, and *Csf1*. **b** In addition, we also measured an increase in the expression of *Flt4*. Data are represented as mean ± SD of six male Wistar rats (*p < 0.05; **p < 0.01; ***p < 0.001)

Since CIH affected the BM vasculature as evidenced by the increased VE-cadherin-expressing vessels, next, we hypothesized the “vascular gene expression” could also be altered. Therefore, we assessed the expression of the so-called “angiocrine genes,” which are expressed by the BM endothelial cells and have been previously shown to be essential for BM recovery following stresses such as irradiation or exposure to chemotherapy [9]. In detail, we sought for molecular correlates to the phenotypic changes observed in the BM microenvironment; that is, genes that could explain the increased erythropoiesis, monocytosis, and vascular changes in the BM microenvironment. As shown in Fig. 5, CIH increased the expression of colony-stimulating factor 1 (*Csf1*), previously shown to modulate monocyte differentiation, proliferation, and survival [63]. Moreover, *Vegfa*, delta-like 4 (*Dll4*), angiopoietin 1 (*Angpt1*) and Fms-like tyrosine kinase 4 (*Flt4*) also increased upon CIH exposure (Fig. 5), suggesting these may be involved in the vascular response observed in the BM of CIH-exposed animals, namely, the increase in VE-cadherin-expressing and SMA-covered BM vessels.

## Discussion

Chronic intermittent hypoxia (CIH) was first reported by Fletcher and colleagues as a model for obstructive sleep apnea [25]. In their protocol, mice were exposed to cycles of intermittent hypoxia for 7 h each day for 35 days and were found to develop a long-term hypertensive response to CIH, as it is observed in OSA patients. Such model fails to reproduce the transient hypercapnia that is observed in OSA patients, determined by airway occlusion, and mimics only the intermittent hypoxic episodes that occur chronically in these patients, allowing us to separate the mechanical component of obstruction from the effect of intermittent hypoxia itself. In fact, the main goal of our work was to explore the effects of CIH by itself in the BM vascular compartment. Additionally, over the years, several other groups have shown that this rat model of CIH mimics many aspects of the obstructive sleep apnea syndrome, such as atherosclerosis and alterations in the hematological parameters (for a review see [16]), and only few have manipulated the CO_2_ levels inside the chambers [19, 36, 58].

It remains unclear whether the partial pressure of CO_2_ in the arterial blood (PaCO_2_) is relevant in humans. Hypercapnia is not a standard parameter analyzed in polysomnographic recordings in patients and therefore there is no consensus on the impact of PaCO_2_ in arterial blood pressure and other parameters in patients with OSA. For instance, in clinical studies of patients with moderate OSA, the changes in PaCO_2_ have seemed to be irrelevant [24] or have shown a slight increase [74] during the apneic events. Combining chronic sustained hypoxia with hypercapnia was shown to restore the subcutaneous PaO_2_ to levels close to the normoxic rats [72] and to induce a smaller increase in the numbers of circulating erythrocytes [60]. However, the effects of combined CIH with hypercapnia in the BM microenvironment and hematopoiesis were not yet described. Therefore, although some data suggest that PaCO_2_ may influence physiological responses to IH, further studies are needed to evaluate the combined effect of IH and hypercapnia. Only male rats were included in our study to avoid hypothetical effects of estrogens on hematologic responses to chronic intermittent hypoxia since it has been described that 17β-estradiol can influence the expression of hypoxia-inducible genes such as VEGF and endothelin-1 [2] and decreases hypoxic induction of erythropoietin gene expression [20, 54, 80].

In this study, we report that CIH induces a deviation from the normal body weight gain observed in normoxia-exposed rats. These results are consistent with those obtained in a parallel study, where the effects of chronic intermittent hypoxia on body weight of male Wistar rats from the NOVA Medical School colony were evaluated. We observed (unpublished data) that rats exposed to 35 days of CIH weighed significantly less compared with age-matched healthy male Wistar rats kept in normoxia. Several authors reported a weight loss of CIH-exposed rats when compared with control rats [69, 70, 75, 85]. Such alteration in body weight might be explained by the production and release of leptin into the circulation as a response to hypoxia [1, 39, 51, 53, 76, 81]. Leptin is coded by an hypoxia inducible gene that acts upon the hypothalamus to control body weight by reducing food intake and increasing energy expenditure [31, 59, 81]. Moreover, leptin-deficient mice exposed to CIH have a normal weight gain, compared to normoxic mice [79], indicating that this is a specific leptin-dependent mechanism rather than just a stress response. In our study, the lower body weight may be due to a higher metabolic activity in the hypoxic rats, as we did not observe significant differences in caloric consumption between the two groups of animals, but this assumption needs further validation.

The increased risk of CIH and OSA patients to develop cardiovascular complications led us to hypothesize that a systemic mechanism might be involved in the pathophysiology of such co-morbidities. The recruitment of bone marrow-derived cells has been amply demonstrated to be involved in the onset and progression of cardiovascular diseases [78]. The involvement of BM cells has, for instance, been shown to be important in the setting of atherosclerosis [28] and has also been implicated in hypertension [78]. Therefore, in the present study, we explored the hypothesis that CIH might affect the BM microenvironment and therefore affect hematopoiesis.

Here, we show a significant increase in BM and PB myeloid (monocyte) counts in animals exposed to CIH, accompanied by an increase in the total monocyte and granulocyte progenitor cell-derived CFUs (CFU-M and CFU-G). Several studies reported an increase in circulating granulocytes both in acute and chronic hypoxia [14, 61] and increased neutrophil and lymphocyte counts in the upper airway mucosa of OSA patients [29, 68]. Interestingly, a significantly lower percentage of macrophages was found in the mucosa of these patients [68]. Nevertheless, chronic intermittent hypoxia increases the amount of pro-inflammatory pulmonary macrophages [56]. No reports of changes in circulating monocyte or in BM myeloid counts as a response to hypoxia exposure were found. However, Roiniotis and his colleagues showed that hypoxia can have a pro-survival effect both in monocytes and macrophages [66] and Yoon described decreased myelopoiesis in *Hif1a*^*−/−*^ embryos [82], suggesting a positive correlation between hypoxia exposure and expansion of the myeloid compartment. Our data also shows that CIH can modulate the BM lymphocyte content, a finding that had not been described either in CIH-exposed rats or OSA patients. Domagala-Kulawik and her colleagues described a decrease in circulating B cells and an increase in several T lymphocyte subsets in OSA patients, but they addressed only peripheral blood and not BM lymphocyte content [17]. Furthermore, our data is consistent with previous reports showing that stabilization of Hif1α and Hif2α in thymocytes resulted in a remarkable increase in thymocyte apoptosis [4, 13] and that HIF1α deficient chimeric mice have impaired B cell development with decreased proliferation of B cell progenitors [38].

In addition, we report an increment in circulating erythrocytes and in blood hemoglobin and hematocrit, which correlates with the higher numbers of erythroid colonies (BFU-E) derived from BM progenitors. This increase in erythroid colonies was also described in rats exposed to chronic sustained hypoxia (4 weeks). However, the authors of that study failed to detect any remarkable alterations in granulocyte-macrophage progenitor numbers [67]. Previous studies have shown that acute and chronic sustained [83] or intermittent hypoxia promoted erythropoiesis [5, 46, 49, 50, 64]. Furthermore, although there is a general lack of healthy controls in most studies, clinical data of OSA patients also suggest an increase in hemoglobin levels [12, 30] and in hematocrit [22, 32], and diurnal variations in erythropoietin levels [10] which together seem to be correlated with the severity of OSA [12, 77]. The effects of hypoxia in platelet parameters are dependent on the hypoxia administration. In detail, short-term chronic sustained hypoxia (1–4 days) was reported to promote thrombocytosis [44]. However, after 4–5 days of exposure, platelet counts returned to normal and thereafter rapidly declined between the fifth and the ninth days of hypoxia, leveling off at half their normal value [44, 48]. Studies in chronic intermittent asphyxia, however, have shown it does not affect platelet count, but instead increases platelet activation and aggregation [18], an effect that is correlated with the severity of the disease in OSA patients [35]. These results deserve further studies but emphasize the differences between sustained and intermittent hypoxia.

One interesting observation in bone marrow sections of animals in CIH was the significant increase in VE-cadherin-expressing vessels and in smooth muscle cell coverage, accompanied by a decrease in the vWF-positive vessels. However, we did not observe an increase in total vessel number assessed by CD105 expression. These findings highlight the heterogeneity of the vascular content of the BM microenvironment (similar findings, in a different context were reported in Remedio et al. 2012) [65] and demonstrate that different vascular markers should be used concomitantly, to avoid misinterpretation of single marker-staining patterns.

These morphological changes in bone marrow vessels upon CIH exposure are also indicative of a molecular process which appears to be favoring vascular stability. vWF is associated with activated and thus less stable vessels, since it is upregulated in endothelial cells treated with FGF2 and VEGF (potent angiogenic inducers) [84]. Contrastingly, VE-cadherin expression and smooth muscle coverage have been associated with increased vessel stability [21], usually induced after an active angiogenic (generating new vessels) process [55]. HIF1α, that we found to be upregulated in hypoxic rats, is one of the major inducers of angiogenesis, as it upregulates *Vegf* expression, ultimately leading to vessel permeability and instability [23, 41, 47, 71]. This process is tightly coupled with a decrease of VE-cadherin in the endothelial tight junctions [37, 47], in a VEGF-dependent manner. Our data suggest that the BM sinusoids may not be responding to the proangiogenic effects of VEGF and instead become more stable upon hypoxia exposure. Additionally, the reported increase in SMA-positive vessels in CIH-exposed rats is suggestive of vessel stabilization. This is in line with the findings that hypoxia promotes endothelial cell activation which will lead to the release of mitogenic factors for smooth muscle cells [33, 52].

An understanding of the role of vessels (and of endothelial cells that comprise them) in organ function and recovery following injury has dramatically changed in the last years. It is now accepted that endothelial cells within each organ express a different subset of trophic growth factors, known as “angiocrine factors,” that will satisfy the function and metabolic demands of that specific organ. Moreover, ECs play an active role in organ recovery, through an adaptation of the expression of these trophic factors, supporting the regeneration and proliferation of stem and progenitor cells in the affected tissues, in a paracrine manner. [8, 9, 57]. In the present study, we reasoned the vascular changes seen in the bone marrows of animals exposed to CIH might affect the production of specific angiocrine factors; identification of such factors could in turn explain the alterations in hematopoiesis seen in CIH animals. We observed significant changes in the expression of *Csf1*, which explains the increase in BM and PB myeloid compartment [11], and in the levels of *Vegfa* and *Angpt1. Vegf* is an hypoxia-inducible gene and is most likely upregulated in response to the increased levels of *Hif1α* in hypoxic rats. Angiopoietin 1 (Angpt1) in particular has been shown to modulate vessel stability by promoting the chemoattraction of smooth muscle cells to newly formed vessels [73], usually in response to augmented VEGF levels [27]. Additionally, Angpt1 protects blood vessels from VEGF-induced permeability by inhibiting internalization of VE-cadherin which leads to an increase in VE-cadherin expression and vessel stabilization [26, 27]. Together, these molecular findings correlate with the vascular changes observed in the bone marrow of animals exposed to CIH.

Taken together, our data obtained from an animal model of OSA, reveal that the systemic effects of CIH result in modulation of the bone marrow microenvironment, namely, the bone marrow vasculature, which in turn might be perturbing hematopoiesis. Our results pave the way for pre-clinical and clinical studies aimed at validating these findings in OSA patients.

## Electronic supplementary material

Below is the link to the electronic supplementary material.

ESM 1 (GIF 13 kb)

High Resolution Image (TIF 995 kb)
