# Supplementary figures and images for: The impact of chronic intermittent hypoxia on hematopoiesis and the bone marrow microenvironment
Source: Pflugers Arch. 2016 Feb 9;468:919–32. doi: 10.1007/s00424-016-1797-6 (PMC4842224; doi:10.1007/s00424-016-1797-6)

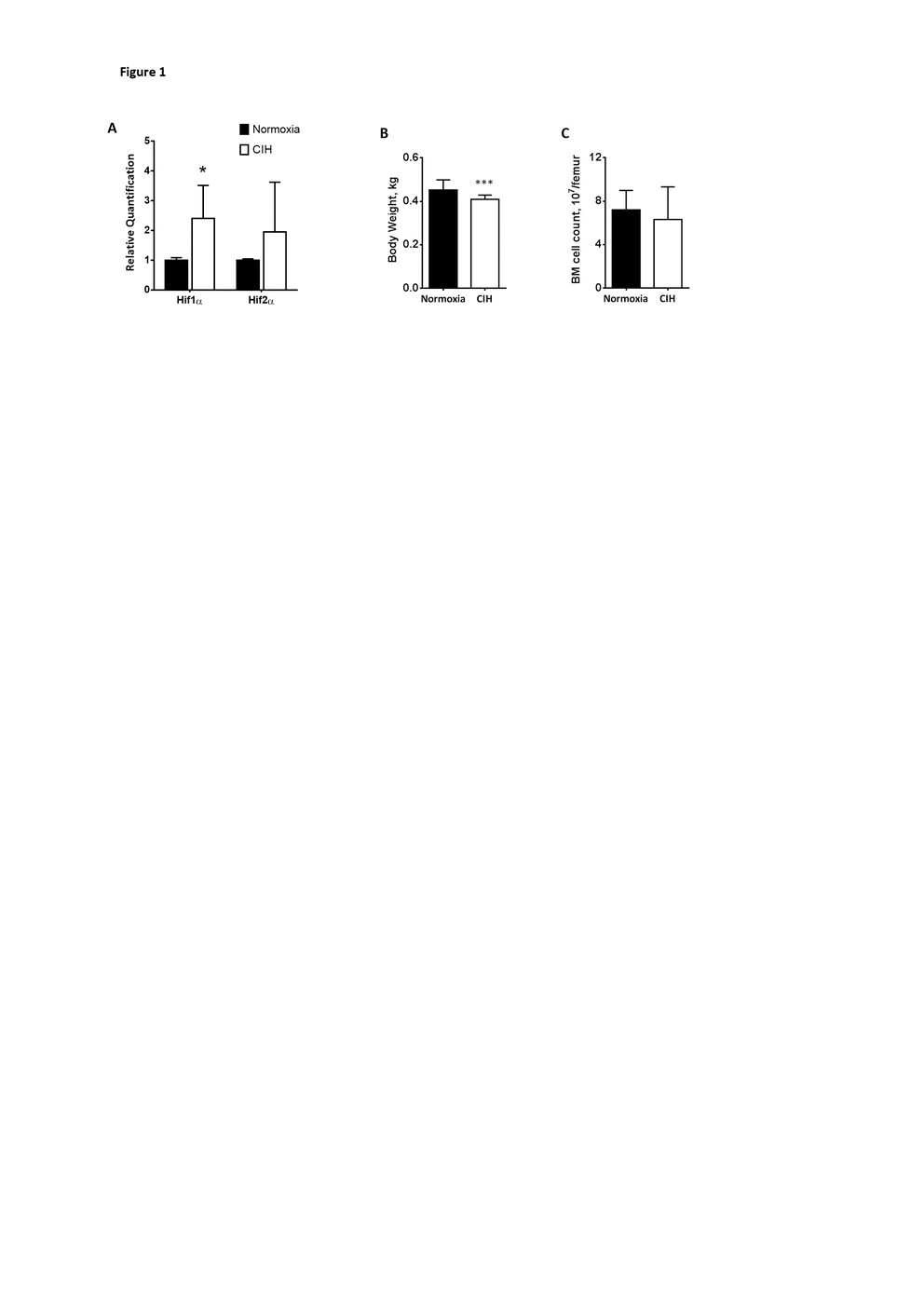

Supplement: Supplementary file 1 — (GIF 13 kb) [file 424_2016_1797_Fig6_ESM.gif]

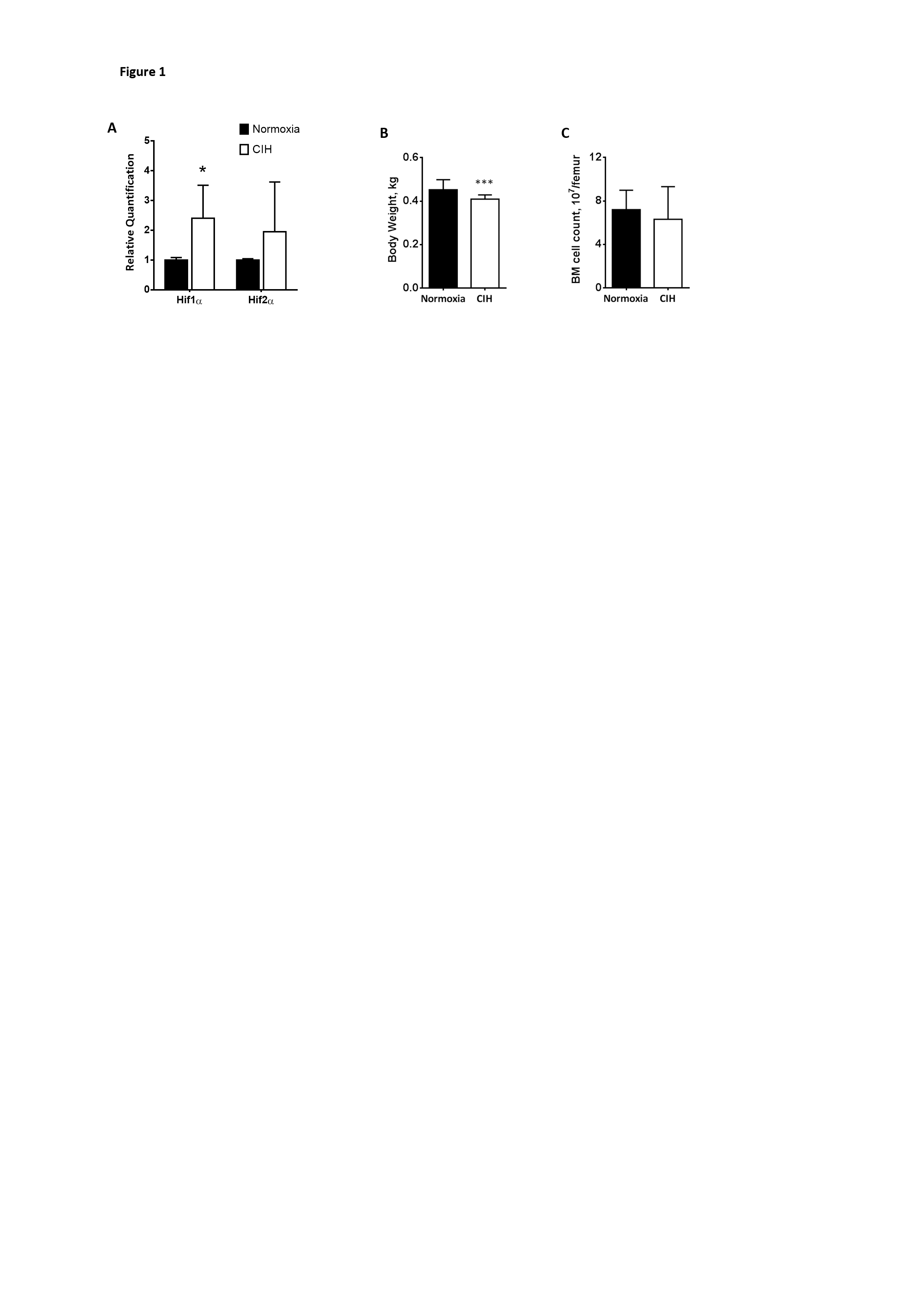

Supplement: Supplementary file 2 — High Resolution Image (TIF 995 kb) [file 424_2016_1797_MOESM1_ESM.tif]
